# Left carotid adventitial vasa vasorum signal correlates directly with age and with left carotid intima-media thickness in individuals without atheromatous risk factors

**DOI:** 10.1186/s12947-015-0014-7

**Published:** 2015-04-17

**Authors:** Maria Vittoria Arcidiacono, Esther Rubinat, Mercè Borras, Angels Betriu, Javier Trujillano, Teresa Vidal, Didac Mauricio, Elvira Fernández

**Affiliations:** Department of Nephrology, Hospital Universitari Arnau de Vilanova, Avda Rovira Roure, 80, 25198 Lleida, Spain; Unitat de Detecció i Tractament de Malalties Aterotrombòtiques, Hospital Universitari Arnau de Vilanova, Avda Rovira Roure, 80, 25198 Lleida, Spain; Institut de Recerca Biomedica de Lleida, University of Lleida, Avda Rovira Roure, 80, 25198 Lleida, Spain; Department of Endocrinology and Nutrition, Hospital Universitari Arnau de Vilanova, Avda Rovira Roure, 80, 25198 Lleida, Spain; Intensive Care Unit, Hospital Universitario Arnau de Vilanova, Avda Rovira Roure 80, 25198 Lleida, Spain; Department of Endocrinology and Nutrition, Hospital Universitari Germans Trias i Pujol, Carretera Canyet S/N, 08916 Badalona, Spain

**Keywords:** Atheromatosis, Vasa vasorum, Contrast-enhanced ultrasound, Carotid arteries, Adventitial layer

## Abstract

**Objective:**

The early identification of the onset of subclinical atheromatosis is essential in reducing the high mortality risk from cardiovascular disease (CVD) worldwide. Although carotid intima-media thickness (cIMT) is the most commonly used early predictor of ongoing atherosclerosis, an experimental model of atherosclerosis, demonstrated that increases in adventitial microvessels (vasa vasorum (VV)) precede endothelial dysfunction. Using the reported accuracy of contrast-enhanced ultrasound (CEU) to measure carotid adventitial VV, this study assessed whether measurements of carotid adventitial VV serve as a marker of subclinical atherosclerotic lesions in a control population with none of the classical risk factors for CVD.

**Methods and results:**

Measurements of cIMT (B-mode ultrasound) and adventitial VV (CEU) were conducted in 65 subjects, 30–70 years old, 48% men, with none of the classical risk factors for CVD. Adventitial VV strongly correlated with its own cIMT only in the left carotid artery. Importantly, the left carotid adventitial VV directly correlated with age.

**Conclusions:**

The increases with age in left carotid adventitial VV in individuals with zero risk for atheromatosis suggest that the measurement of carotid adventitial VV could be an accurate and sensitive marker for the diagnosis of subclinical atheromatosis and therefore a prominent tool for monitoring the efficacy of anti-atheromatous therapies.

## Introduction

Atherosclerosis is the leading cause of death in the general population worldwide [1]. The ability to identify the atherosclerotic process at early stages is of great value for attenuating the risk of adverse cardiovascular disease (CVD) events.

At present, carotid intima-media thickness (cIMT) is the earliest and most widely used predictor of atheromatosis. Indeed, not only is cIMT associated with several traditional risk factors including smoking, high cholesterol, high blood pressure, and high glucose [2,3], but more significantly, cIMT improves the prognostic risk prediction of either one or more than one of any of these traditional risk factors, particularly in women [4]. Importantly, ultrasonographic measurements provide an accurate estimation of cIMT. To this point, Pignoli et al. find no significant differences between the ultrasonographic measurements of the IMT in the common carotid arteries and those determined histologically [5].

Important for the identification of the pathophysiology underlying cIMT was the immunohistochemical analysis of human carotid atherosclerotic lesions. At pre-atheroma stages, microvessels originating from the adventitial layer are present in the intima layer, while they are absent in the normal intima and also in atheromatous lesions that are independent of intimal thickening [6,7]. These microvessels are known as vasa vasorum (VV), and they are physiologically located in the adventitial layer providing nourishment to medium and large vessels including aorta, coronary, femoral, and carotid arteries. Experimental evidence supports a role of the neoangiogenesis originating from the adventitial layer in the initiation and progression of the atherosclerotic process [8]. This initial demonstration that hypercholesterolemia increases adventitial VV before the development of atherosclerotic lesion in porcine coronary arteries is further supported by the studies by Herrmann et al., using three-dimensional micro-computed tomography (CT) [9]. In the coronary arteries of pigs fed a hypercolesterolemic diet, the marked increases in the density of adventitial VV precede endothelial dysfunction, the first alteration leading to atherosclerosis [9]. These experimental findings suggest that imaging VV in the adventitia *in vivo* could identify stages in the development of atherosclerosis prior to the well-recognized increases in cIMT, thus favoring an early identification of patients with a higher risk of cerebrovascular events.

Contrast-enhanced ultrasound (CEU) has been introduced as a useful, non-invasive and inexpensive technique for studying VV in the carotid arteries [10]. Indeed, several studies have identified plaque neovascularization using CEU, which were histologically confirmed after endarterectomy [11-13]. However, these studies were prevalently focused on intraplaque neovascularization. In order to use the degree of neovascularization necessary for CEU assessment to predict cerebrovascular risk, a better understanding of the physiological/pathological amount of VV in the carotid adventitial layer was necessary. Indeed, the first report of an increase of the periadventitial VV network in patients with carotid stenosis of at least >50%, compared with that in patients with no carotid stenosis, utilized a combination of sonographic contrast agent and B-flow images [14]. More recently, the direct *in vivo* visualization of the adventitial neovascularization by CEU has been reported: values from type 2 diabetic patients showed a higher adventitial vascularization in comparison with the values from healthy subjects. Furthermore, diabetic patients affected by retinopathy showed a higher adventitial VV than did patients not affected by retinopathy [15].

Therefore, the aim of the study was to study a group of healthy volunteers with none of the classical risk factors for CVD (smoking, hypertension, hypercholesterolemia, diabetes) to assess the physiological density of adventitial VV.

## Materials and methods

### Study subjects

Subjects with none of the classical risk factors for cardiovascular disease were enrolled to obtain a physiological estimate of adventitial VV content prior to the development of atheromatosis. All of the 65 enrolled subjects were non-smokers, and with morphometric and biochemical parameters within the normal range. The age range was between 30 and 70 years, and 48% were men. During enrollment, some subjects were excluded for the following reasons: 1) They met the exclusion criteria for the administration of the contrast agent including: a) recent cardiac instability, b) recent (<7 days) coronary intervention, c) class III or IV heart failure, d) severe pulmonary hypertension, or e) allergic reaction to sulphur hexafluoride, the main component of the contrast agent; 2) The detection of a carotid plaque during B-mode ultrasound. This protocol was approved by the ethical committee at the University Hospital Arnau de Vilanova (HUAV, Lleida, Spain). After being informed of the goals and protocols, all subjects signed consents before morphometric parameter acquisition and the drawing of blood.

### Morphometric and biochemical parameters

Morphometric parameters were obtained by standard methods. Briefly, weight and height were measured with a digital weight scale with a stadiometer. Body mass index (BMI) was then calculated as weight in kilograms divided by the square of height in meters. The waist circumference (WC) was measured in centimeters at the umbilicus level. Blood pressure (BP) was measured as the average of triplicate values obtained with an automated oscillometer (Omron HEM-705CP) while patients were seated and had rested for ten minutes. Pulse pressure (PP) was calculated as the difference between mean systolic (SBP) and mean diastolic blood pressure (DBP). Biochemical parameters (glucose, total cholesterol (Cholesterol), HDL and LDL cholesterol, and triglycerides (TG)) were obtained after overnight fasting using the standard methods of the laboratory of Clinical Biochemistry at the HUAV. Ultrasensitive C-reactive protein (CRP) was measured in the same laboratory using an immunoturbidimetric assay (Roche Diagnostic on a Hitachi automated analyzer).

### Standard and contrast-enhanced carotid ultrasound

All subjects (supine position) underwent a B-mode ultrasound examination of the extra-cranial carotid arteries including the common, bulb, internal, and the external carotid artery (CA). A prior axial exploration was followed by a longitudinal exploration for the evaluation of atheromatous plaque presence and for measurements of cIMT of the far wall of the common artery. The cIMT was calculated as the gray-scale layer comprised within the luminal blood and the carotid adventitia layer at the level of 1 cm proximal to the bifurcation. Two independent readers (MVA, TV) read cIMT and reproducibility, according to the obtained intraclass correlation coefficient (ICC) of 0.822 (range 0.745-0.877), was classified as good (range 0.7-0.9). Next, subjects underwent the CEU imaging procedure using the contrast agent Sonovue (Bracco Spa, Milan, Italy), a phospholipidic shell containing sulfure hexafluoride. A Sonovue vial was solubilized in 5 ml of saline, and a 2.5 ml bolus was injected first, followed by 10 ml of a saline flush, in the antecubital vein for each CA explored (20-gauge needle to avoid microbubbles rupture). According to the manufacturer’s information, the contrast agent has no adverse effects at this dose and was sufficient to obtain a strong and clear signal for a 1-minute image recording. The CEU imaging was performed with a Siemens Sequoia 512 using the 15L8W linear array probe (7 Mhz) with a low mechanical index of 0.18. The Sequoia 512 is equipped with Cadence contrast Pulse Sequencing (CPS) technology able to combine the nonlinear fundamental and higher harmonic nonlinear fundamental contrast signals determining a high sensitivity and specificity of contrast agent detection. During imaging acquirement it is possible to specifically separate “tissue only”, “contrast agent only” or “both together”, specifically we worked on “CA” methodology that means “contrast agent only”. All the videos were stored for a posterior reading using the Siemens software Syngo. As previously described [15], adventitial VV content in the far adventitial layer was calculated as the average of the ratios of the intensities in the 2 mm above the intima-lumen boundary and the intensities of the 2 mm below the media-adventitia boundary of the common carotid artery 1 cm proximal to the bifurcation. The final result (VV signal) was calculated as the average of 10 to 20 ratios calculated for each diastolic frame in which both the lumen intensity and the adventitial intensity was high and stable within a 1 minute video recording. As reported [15], the intraobserver variability was classified as very good (CCI = 0.930; 0.84-0.970); while, the reproducibility of the method, calculated on two readers (AMV, RE), was classified as good (ICC = 0.870; 0.780-0.920), comparable to the reproducibility of cIMT measurements. As reported in [16] we excluded from reading videos in which signals were observed in the far arterial wall before contrast injection therefore avoiding artefact images. Additional reasons for reading exclusion were: 1) the contrast agent disappeared rapidly thus impeding to be properly visualized; or 2) the presence of an ultrasound shadow in the area of the carotid under analysis that impeded the reading**.**

### Statistical analysis

Data analysis was performed by an investigator totally blinded to the characteristics of the patients. The SPSS v17 software was used for analysis. Morphometric and biochemical values, as well as cIMT and VV ratio, were expressed as mean ± standard deviation if there was no significant deviation from normal distribution, or as median (interquartile rage) if non-normal distribution was observed. Normal distribution was analyzed using the Kolmogorov-Smirnov test. The Mann–Whitney test examined the statistical difference of the parameters in the two genders. The Spearman’s rank correlation coefficients assessed the correlation between the adventitial VV ratio and the cIMT and between the VV ratio and the biochemical or anthropometric parameters. Statistical significance was defined as p < 0.05.

## Results

Subject characteristics, stratified by gender, are shown in Table 1. According to the inclusion criteria, age distribution was similar between women (50 ± 10) and men (48 ± 11), and all examined parameters were within the normal physiological range. Gender differences were observed for serum HDL and LDL levels (women: 58.8 ± 11 vs men: 49.1 ± 12, p = 0.003, and women: 106.3(94–120) vs men: 122.6(97–136), p = 0.032, respectively), and, as expected, the waist circumference (WC) was lower in women than in men (85.7 ± 10 vs 91.9 ± 8, p = 0.009, respectively).

**Table 1:** **Baseline characteristics of the study population**

	**Female (n = 34)**	**Male (n = 31)**	***p-value***
**Age (years)**	50 ± 10	48 ± 11	*0.626*
**Glucose (mg/dl)**	91.7 ± 10	93.9 ± 7	*0.092*
**Cholesterol (mg/dl)**	177.3 ± 26	181.4 ± 27	*0.275*
**HDL-Cholesterol (mg/dl)**	58.8 ± 11	49.1 ± 12	***0.003***
LDL- Cholesterol (mg/dl)*	106.3 (94–120)	122.6 (97–136)	***0.032***
TG (mg/dl)*	56.0 (48–68)	65.0 (47–90)	*0.205*
CRP (mg/l)*	0.8 (0.5-1.7)	0.8 (0.5-2.3)	*0.568*
**SBP (mmHg)**	120.3 ± 14	124.4 ± 11	*0.106*
**DBP (mmHg)**	74.5 ± 8	74.9 ± 8	*0.989*
**PP (mmHg)**	45.8 ± 10	48.6 ± 9	*0.264*
**WC (cm)**	85.7 ± 10	91.9 ± 8	***0.009***
**BMI (Kg/m2)**	24.8 ± 4	25.2 ± 2	*0.290*

*Identified variables with non-normal distribution. Values of these variables are described with Median (IQR). For variables with no significant deviations from normal distribution, values are provided as mean ± standard deviation. Mann–Whitney test p-values to test differences between females and males are provided given the small sample size. Statistically significant p-values are indicated in bold.

TG: triglycerides; CRP: C-reactive protein; SBP: systolic blood pressure; DBP: diastolic blood pressure; PP: pulse pressure; WC: waist circumference; BMI: body mass index.

Ultrasound measurements of cIMT and of adventitial vasa vasorum (VV) content are shown in Table 2. As shown in panel A, the mean cIMT value of the common CA was 0.646 ± 0.107, and no difference was observed between women and men (0.636 ± 0.107 vs 0.656 ± 0.108). The mean VV ratio (panel B) was 0.577 ± 0.119 and there was no difference in VV ratio between women and men (0.560 ± 0.104 vs 0.594 ± 0.133). In addition, there were no statistical differences between cIMT and VV ratio in the right and the left CA of both, women and men.

**Table 2:** **cIMT and carotid adventitial VV values**

**A**			
	**cIMT (mm)**	**Right cIMT (mm)**	**Left cIMT (mm)**
**Female**	0.636 ± 0.107	0.626 ± 0.102	0.646 ± 0.130
**Male**	0.656 ± 0.108	0.641 ± 0.127	0.671 ± 0.127
**Females vs. Males** ^*^	0.340	0.608	0.412
**Total**	0.646 ± 0.107	0.633 ± 0.114	0.658 ± 0.128
**B**			
	**VV (ratio)**	**Right VV (ratio)**	**Left VV (ratio)**
**Female**	0.560 ± 0.104	0.568 ± 0.130	0.544 ± 0.146
**Male**	0.594 ± 0.133	0.598 ± 0.208	0.596 ± 0.144
**Females vs. Males** ^*^	0.162	0.403	0.270
**Total**	0.577 ± 0.119	0.582 ± 0.171	0.573 ± 0.146

Values are expressed as mean ± standard deviation after confirming no significant deviation from the normal distribution. (*) Mann–Whitney test p-values to test differences between females and males are provided given the small sample size.

**Figure 1:**
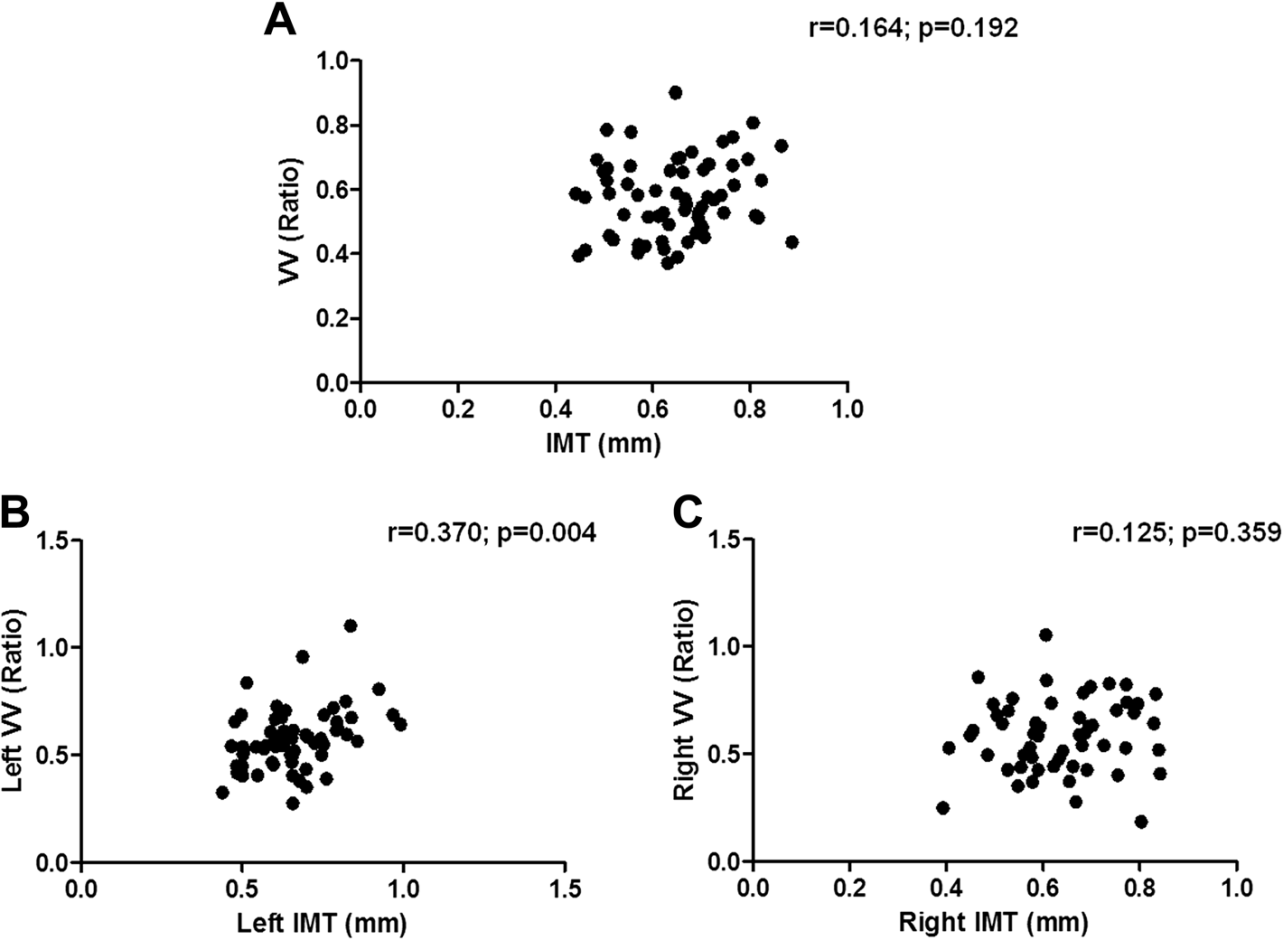
cIMT correlates directly with adventitial VV only in the left carotid artery. Panel **A**. Average of left and right values for cIMT and adventitial VV. Panels **B** and **C**. Left and right values of cIMT and adventitial VV. r = Spearman’s correlation coefficient. Statistical significance is defined as p < 0.05.

As shown in Figure 1A, no correlation was observed between the average left and right cIMT and the average left and right of adventitial VV ratio of all 65 subjects. Interestingly, although no correlation was observed between the right cIMT and the right carotid adventitial VV (r = 0.125, Figure 1 panel C), the left cIMT significantly correlated with the left carotid adventitial VV (r = 0.370, p = 0.004, Figure 1 panel B).

Moreover, as shown in Table 3, only age correlated with the left VV ratio (r = 0.313, p = 0.015). None of the biochemical or anthropometric parameters examined correlated with the adventitial VV ratio, including when separately considering the right and the left CA.

**Table 3:** **Correlation between carotid adventitial VV ratio and the biochemical or anthropometric parameters**

	**Right VV Ratio**	**Left VV Ratio**
**Age (years)**	−0.079 (0.563)	0.313 (0.015)^*^
**Glucose (mg/dl)**	−0.129 (0.344)	−0.150 (0.252)
**Cholesterol (mg/dl)**	0.135 (0.321)	−0.144 (0.272)
**HDL-Cholesterol (mg/dl)**	0.037 (0.787)	0.035 (0.789)
**LDL-Cholesterol (mg/dl)**	0.063 (0.643)	−0.083 (0.530)
**TG (mg/dl)**	0.147 (0.281)	−0.139 (0.291)
**CRP (mg/l)**	0.026 (0.850)	−0.188 (0.150)
**SBP (mmHg)**	−0.007 (0.961)	0.115 (0.383)
**DBP (mmHg)**	0.042 (0.762)	0.038 (0.773)
**PP (mmHg)**	−0.079 (0.566)	0.138 (0.296)
**WC (cm)**	0.172 (0.228)	0.038 (0.785)
**BMI (kg/m** ^**2**^ **)**	0.018 (0.891)	−0.074 (0.593)

Spearman’s correlation coefficient (p-value). (*) p < 0.05.

TG: triglycerides; CRP: C-reactive protein; SBP: systolic blood pressure; DBP: diastolic blood pressure; PP: pulse pressure; WC: waist circumference; BMI: body mass index.

## Discussion

This study in healthy individuals with no classical risk factors for atheromatosis, in which exclusively the left carotid adventitial VV density correlated with both age and left cIMT, supports the accuracy and sensitivity of measurements of the left carotid adventitial VV density as an early marker of subclinical atheromatosis.

Indeed, using a population without clinical atheromatosis and with none of the classical risk factors for the development of atheromatous disease, a statistical significant correlation between the left cIMT and the left VV ratio was observed (r = 0.370; p = 0.004). No correlation was observed in the right side, or when considering the VV ratio and the cIMT as the average of the values of the left and the right carotid arteries. The latter finding is similar to that reported by Sampson et al. in patients with minimal atherosclerosis, which demonstrates no association between cIMT and adventitial VV when considering the average of both CA (left and right) [17]. Therefore, the results of the current study highlight the importance of examining the left and the right CA independently in patients not affected by atheromatosis.

Until now, most clinical studies have focused on the contribution of increases in neovascularization (vasa vasorum) to plaque instability [18]. Furthermore, the few post-mortem studies in humans showing increases in VV in the plaque are insufficient to support the contribution of increases in adventitial VV to initiate the atherosclerotic process that was observed in experimental hypercholesterolemic settings [9]. The direct correlation between the left VV ratio and the left cIMT demonstrated herein, and our recent demonstration that, despite similar values of cIMT, diabetic patients with retinopathy (angiogenesis) presented a higher carotid adventitial VV ratio [15] support the experimental findings in which increases in adventitial VV precede the increases in cIMT [9].

The decision to distinguish between the VV density in left and right CAs was based upon Lemne’s findings of higher values of the cIMT on the left CA in normotensive individuals [19] and more importantly, by the fact that the association between cardiovascular risk and vascular anatomy is not uniform for the thickness of intima-media layer [20] or for the presence of plaque [21,22]. Furthermore, this anatomical difference is attenuated with the occurrence of atherosclerotic risk factors such as hypertension. Indeed, subjects with mean blood pressure of 90 mmHg (or lower) had a higher cIMT in the left than in the right CA, while, when mean arterial blood pressure was higher than 90 mmHg, not only did age not affect cIMT, but also the differences in wall thickening between the left and the right CA attenuated [23]. These findings are in line with the Rotterdam Study in which, in a population aged 55 or over, the differences between participants with and without hypertension are significant only in the right CA, therefore suggesting the possibility that atherosclerotic lesions develop in the left side earlier than in the right side [24]. A potential explanation for this difference is that the left CA originates directly from the aortic arch and is therefore exposed to a constantly higher shear stress [25]. The fact that atherosclerotic lesions appear earlier in the left than in the right CA could in part explain our finding of a correlation between the adventitial VV and the cIMT only in the left CA.

None of the measured biochemical or anthropometric parameters, all of which are highly involved in the development of atheromatous disorders, except for age correlated with adventitial VV ratio. Since aging is the natural determinant of a higher prevalence of atheromatous lesions in otherwise healthy individuals, our demonstration of no association of the right adventitial VV with age supports the high sensitivity of CEU measurements of left adventitial VV density for the diagnosis of subclinical atheromatosis.

A limitation of the present study is the small number of enrolled patients due to the strict inclusion criteria. Furthermore the strong direct correlation between age and left adventitial VV in this population with zero risk for atheromatosis, underscores the need to conduct a study with similar inclusion criteria but with the appropriate statistical power to obtain reference values of VV density in the left and right carotids within a gender and also at different ranges of age. Importantly, the present study provides the mandatory data to infer the appropriate sample size to accurately establish physiological values of adventitial VV density. This is a mandatory first step to evaluate the abnormalities in adventitial VV in patients at a higher risk for cardiovascular disease.

## Conclusions

In conclusion, our findings suggest that CEU measurements of carotid adventitial VV provide a novel marker to explore the atheromatous development process and in monitoring disease progression and the response to anti-atheromatous strategies. Additional studies in populations considered at a higher CV risk, including smokers or individuals with hypertension, hypercholesterolemia, diabetes, or renal disease are needed to further explore the distinct contribution of increases in left or right adventitial VV ratio to the progression of the atheromatous process and to its regression in response to anti-atheromatous therapies.
